# Risk Factors for Relapse of Schizophrenia in the Elderly During the Maintenance Phase: A Matched Case-Control Study

**DOI:** 10.31083/AP39866

**Published:** 2025-04-21

**Authors:** Biqi Zu, Ting Wang, Chunying Pan, Wentao Li, Libin An, Juan Yin, Yulan Wu, Junting Xu, Dandan Li, Xin Wu, Ziwei Xie

**Affiliations:** ^1^School of Nursing, Dalian University, 116001 Dalian, Liaoning, China; ^2^Nursing department, Dalian Seventh People’s Hospital, 116023 Dalian, Liaoning, China

**Keywords:** elderly, maintenance period, psychiatry, schizophrenia, recurrence

## Abstract

**Objective:**

To investigate the risk factors for relapse among elderly schizophrenia patients undergoing maintenance phase treatment, aiming to offer insights for relapse prevention in this population.

**Methods:**

A survey was conducted of elderly schizophrenia patients in the maintenance phase who attended outpatient clinics at a specialized psychiatric hospital from October, 2021 to September, 2023. The survey included both general and clinical data. Univariate analysis and multivariate non-conditional logistic regression analysis were conducted to identify independent risk factors for relapse in elderly schizophrenic patients undergoing maintenance phase treatment. A receiver operating characteristic (ROC) curve was drawn based on logistic regression results and the area under the curve (AUC) was used to evaluate the predictive value of each risk factor for relapse studied in these patients.

**Results:**

A total of 247 patients were collected, with 225 patients included in the analysis: 75 in the recurrence group and 150 in the non-recurrence group. Multivariate logistic regression analysis indicated: Irregular medication status (odds ratio (OR) = 3.302, 95% confidence interval (CI): 1.386–7.871), low exercise frequency (OR = 2.770, 95% CI: 1.141–6.726), family care points (OR = 0.647, 95% CI: 0.514–0.813), life event points (OR = 1.353, 95% CI: 1.194–1.533), and sleep duration (OR = 0.630, 95% CI: 0.504–0.788) as independent influencing factors for relapse during the maintenance phase of elderly patients with schizophrenia. The AUC for predicting relapse varied among these factors: Medication status (AUC: 0.660, 95% CI: 0.594–0.726), exercise frequency (AUC: 0.663, 95% CI: 0.599–0.727), family care (AUC: 0.691, 95% CI: 0.618–0.764), life events (AUC: 0.792, 95% CI: 0.731–0.853), and sleep duration (AUC: 0.789, 95% CI: 0.718–0.859). When considering all influencing factors, the AUC for predicting relapse during maintenance phase treatment of elderly patients with schizophrenia was 0.908 (95% CI: 0.867–0.949).

**Conclusion:**

Medication status, exercise frequency, family care, life events and sleep duration emerged as independent influencing factors for relapse among elderly schizophrenia patients during maintenance phase treatment. Paying attention to these influencing factors simultaneously is suggested to prevent recurrence.

## Main points

1. **Identification of Independent Relapse Risk Factors**: The study identified five independent risk factors for relapse, including irregular medication (OR = 3.302), low exercise frequency (OR = 2.770), inadequate family care (OR = 0.647), negative life events (OR = 1.353) and shorter sleep duration (OR = 0.630). These factors play a significant role in preventing relapse during the maintenance phase in elderly schizophrenia patients.

2. **Superiority of the Combined Predictive Model**: ROC curve analysis showed that a combined predictive model, which incorporates all identified risk factors, has a superior predictive value (AUC = 0.908) compared to individual risk factors. This indicates that considering multiple risk factors collectively more accurately predicts relapse risk in elderly schizophrenia patients.

3. **Importance of Multidimensional Management**: Study results highlight the importance of adopting a multidimensional management strategy in the maintenance treatment of elderly schizophrenia patients. By comprehensively addressing irregular medication, low exercise frequency, inadequate family care, negative life events and shorter sleep duration, relapse prevention can be more effectively achieved.

## 1. Introduction

Schizophrenia (SCZ) encompasses a collection of severe and recurrent mental disorders with unknown etiology [1]. The complete remission rate for SCZ is only 20%, highlighting its high propensity for recurrence [2]. Each relapse results in irreversible damage to the integrity of brain ventricles [3], compromises social functioning and diminishes overall quality of life [4], thereby escalating the economic burden on both families and society [5, 6]. According to the 2021 Global Burden of Disease report, approximately 3.89 million SCZ patients are aged 60 or older [7]. With rapid aging of the population, the overall burden of SCZ is expected to continue to increase [8]. Diminished disease resistance and post-disease recovery capabilities of an elderly population renders them more susceptible to the adverse effects of relapse in SCZ.

The maintenance phase, following acute and consolidation therapy (as per the clinical practice guidelines [9] mentioned: Stage 4, severe, persistent and unremitting course), may extend throughout a patient’s lifetime. Unlike other stages, patients in the maintenance phase are often outside the hospital setting, where numerous factors impinge upon disease stability, lead to recurrent episodes [10, 11]. Concurrently, expert consensus, guidelines and research [1, 12, 13] underscore that the maintenance period is pivotal for averting disease relapse. Nevertheless, systematic inquiry reveals few studies probing the factors influencing relapse in elderly SCZ patients during the maintenance phase.

Given the rapid acceleration of population aging, it is crucial to implement targeted measures to address relapse among elderly SCZ patients undergoing maintenance phase treatment. This study aims to investigate the risk factors associated with relapse among such patients and offers guidance and reference for preventing relapse in this demographic.

## 2. Methods

### 2.1 Study Design

To mitigate confounding factors, a matched case-control study was devised that utilized hospital information systems. Patients meeting the inclusion and exclusion criteria were matched chronologically, in a 1:2 ratio between the recurrence (case) and non-recurrence (control) groups based on age (±2 years) and disease duration (±2 years). Previous studies have underscored age [14] and disease duration [15] as potential influencing factors for recurrence. Given the older age and prolonged disease duration of the study cohort, sampling bias was reduced by matching based on age and disease duration.

### 2.2 Study Setting

The research, including applications, ethical review and data collection was conducted at a hospital from December 2023 to May 2024. Situated in the northeast of China, the hospital is a tertiary psychiatric and psychological hospital and serves as the teaching hospital for a university school. The hospital handles over 200,000 outpatient visits annually, addressing the mental health needs of over seven million people in the city and its surroundings.

### 2.3 Study Participants

Elderly patients with SCZ admitted to the hospital from October 2021 to September 2023 were selected as the study participants. Inclusion criteria: (1) Meeting the International Classification of Diseases-10th edition (ICD-10) diagnostic criteria for SCZ (F20) in ICD-10; (2) Age ≥60 years; (3) In the maintenance period. Exclusion criteria: (1) Combination with other severe mental or neurological disorders or physical illness; (2) Patient data was incomplete or unavailable.

Guidelines and The Positive and Negative Syndrome Scale (PANSS) [16] were used to maintain baseline balance: When the patient’s condition remains unchanged for at least 6 months during the consolidation phase [17], and the following items have scores of less than 3 (mild): P1 delusion, P2 conceptual disorder, P3 hallucination, and P6 suspicion/persecution, the patient is considered to be in a stable condition and can transition to the maintenance phase [18, 19].

### 2.4 Grouping Criteria

Based on presence or absence of recurrence. Any of the following criteria were considered as recurrence: (1) PANSS total score increased by ≥25% from baseline value (or score ≥10 when baseline PANSS total score ≤40) [20]; (2) Major manifestations included escalating the dosage and type of antipsychotics in response to disease fluctuations, increasing the frequency of visits or hospitalizations due to disease fluctuations and intensifying symptom management to prevent accidents or hazards [21].

### 2.5 Study Size

PASS software version 2021 (NCSS, LLC, Kaysville, Utah, USA) was used for sample size calculation. Utilizing the sample size calculation method for multivariable logistic regression, a total of nine variables were included in the model (eight controlled variables and one tested variable). Setting α at 0.05, power at 0.90, and f^2^ at 0.05, the required sample size was calculated to be 224 cases. Considering a 10% loss to follow-up or data missing, the required sample size was adjusted to 247. Recurrence and non-recurrence groups were matched by age (±2 years) and disease duration (±2 years) in a 1:2 ratio. After data matching, a total of 225 participants were included. With an α level of 0.05, the test power was 0.902.

### 2.6 Research Tool

#### 2.6.1 General and Clinical Data Questionnaire

The questionnaire on general and clinical data was designed by the researchers after reviewing domestic and international literature. It included various parameters such as patient demographics (gender, age, body mass index (BMI), disease duration, educational status, marital status), family genetic background, smoking and drinking status, premorbid personality, sleep duration, exercise frequency, household registration type, per capita monthly income, payment method, family care, social support and life events. Clinical data included medication status and adverse drug reactions.

**Definition Criteria for General and Clinical Data: BMI**: Calculated as weight (in kilograms) divided by height squared (in meters). The study designated it as a continuous variable. **Family Genetic Background**: Defined as the presence of mental illness across two lines and three generations within the family lineage [1]. **Smoking Status**: Classified as individuals smoking at least one cigarette per day for at least six months, with all others categorized as non-smokers [22]. **Drinking Status**: Regardless of the type of alcoholic beverage, consuming at least 50 g per occasion and at least once a week is considered drinking, while occasional or no consumption is classified as non-drinking [22]. **Exercise Frequency**: High frequency is defined as engaging in exercise at least once per week with a total weekly duration exceeding 60 minutes within the past month. Low frequency refers to individuals who do not meet these criteria. **Per Capita Monthly Income**: Grouped based on data from the City Bureau of Statistics [23] and household registration type. **Medication Status**: Classified as regular or irregular, with irregular including instances of missing medication or hiding medicine use within the past month [24]. **Drug Adverse Reactions**: Regardless of whether these reactions were long-standing or occurred during the observation period. Included extrapyramidal and cardiovascular adverse effects such as excessive sedation, gastrointestinal symptoms, salivation, weight gain, abnormal liver function, malignant syndrome, blood system changes and severe electrocardiogram alterations [24].

#### 2.6.2 Family Apgar Index Scale

The scale [25] is used to measure individual satisfaction with family functions, including: Fitness, cooperation, growth, emotional degree and affinity density five dimensions, each item in “almost rarely”, “sometimes”, “often”, is scored zero, one, or two points, respectively. Total score ranged from zero to ten, with seven to ten representing good family functioning, four to six representing moderate family functioning impairment, and zero to three representing severe impairment of family functioning.

#### 2.6.3 Social Support Rating Scale, SSRS

The scale [26] consists of 10 items in three dimensions: subjective support (four items), objective support (three items) and support utilization (three items). The total score is the sum of the score of each item (13–66 points). SSRS score <20 were classified as less social support, SSRS score ≥20 were classified as normal, scores 20–30 were classified as general social support, with 31–40 classified as satisfactory social support.

#### 2.6.4 Life Events Scale

The scale [27] accurately reflects positive/negative events and their duration. The scale includes 48 common life events (involving family life, work, study, social life, etc.) and two blank items for unlisted in the table. The life events experienced by each patient were investigated, asking whether they occurred, when they occurred, the nature of the event, the degree of mental impact of the event and the duration of the impact. This study refers to the negative events.

### 2.7 Data Sources and Bias Control

All included patients entered the maintenance phase after consolidation phase treatment (six months later) and began a six-month observation period. During this period, information from monthly outpatient visits was recorded in the medical record system. Patients were grouped based on whether they experienced relapse and data were collected accordingly. For the relapse group, data were retrospectively reviewed up to the point of relapse (which could be less than six months). For the non-relapse group, data is collected at the end of the observation period. Patients with less than 5% missing data underwent supplementary data collection through interviews with family members or patients after assessing their emotional stability; patients with missing data exceeding 5% were excluded. To enhance the quality and validity of data collection, a preliminary survey was conducted on 5% of participants beyond the sample size (12 individuals) and the data collection protocol was refined based on the survey results. Evaluators, data collectors and supervisors underwent a three-day training session on the consistency of data collection procedures. During the data collection phase, information collectors were blind to patient enrollment, with evaluators assigning groups and informing collectors for data collection completion. The principal investigator examined the completeness and consistency of collected data and performed case matching based on matching principles. Additionally, completed questionnaires were coded and double data entry and verification were conducted to prevent errors during the data entry process.

### 2.8 Statistical Methods

Normally distributed continuous data were presented as mean ± standard deviation, and differences between groups were compared using the* t-*test. Categorical data were presented as frequencies and proportions and differences between groups were assessed using the *chi-square* test. Non-normally distributed continuous data were described as median (*p*_25_, *p*_75_) and differences between variables were compared using the *Wilcoxon rank-sum* test. Factors with statistical differences in univariate analysis (*p* < 0.05) were included in the multivariate logistic regression analysis using the enter method, with α set at 0.05 for entry and 0.10 for removal, to identify the influencing factors for relapse among elderly SCZ patients during maintenance phase treatment. SPSS software version 26.0 (IBM Corporation, Armonk, New York, USA) was used for analysis, and R software version 4.3.3 (R Foundation for Statistical Computing, Vienna, Austria) was used to plot ROC curves and forest plots. The AUC was calculated to compare the predictive efficacy of each influencing factor for relapse among elderly SCZ patients during maintenance phase treatment. All tests were two-tailed, and *p* < 0.05 was assumed to indicate statistically significant differences.

## 3. Results

### 3.1 General Information

In this study, data from 247 patients were collected. Following matching based on age (±2 years) and disease duration (±2 years) (Table 1), statistical analysis encompassed 225 matched patients, comprising 75 cases in the recurrence group and 150 cases in the non-recurrence group. Twenty-three patients experienced matching failure and were excluded from the analysis. Of the total sample, 127 (56.4%) were male and 98 (43.6%) were female. The mean age was 68.96 ± 5.53 years, with a disease duration of 32.82 ± 6.76 years. Urban and rural residence distribution comprised 114 (50.7%) and 111 (49.3%) cases, respectively. Recurrence occurred in 75 (33.3%) cases, while 150 (66.7%) cases did not experience recurrence. The observation duration for the relapse group was 4 ± 1.7 months, while it was six months for the non-relapse group.

**Table 1.** **Patient-matching characteristics**.

Matching factors	Recurrence (*n *= 75)	Non-recurrence (*n *= 150)	Statistic	*p*-value
Age (years)	67.0 (61.0, 73.0)	69.0 (65.0, 74.0)	–1.100	0.271
Illness duration (years)	25.8 (20.3, 31.4)	25.9 (20.2, 31.1)	–0.667	0.505

### 3.2 Single Factor Analysis

Following variable assignment and quantization (Appendix Table 5), single factor analysis was conducted. Significant differences (*p* < 0.05) were observed in nine variables (Table 2): gender (*p *= 0.001), alcohol consumption (*p *= 0.021), medication (*p* < 0.001), exercise frequency (*p* < 0.001), drug adverse reactions (*p *= 0.001), family care (*p* < 0.001), social support (*p *= 0.008), life events (*p* < 0.001) and sleep duration (*p* < 0.001).

**Table 2.** **Univariate analysis of factors affecting relapse in elderly schizophrenia patients during maintenance phase**.

Variables		Recurrence (*n *= 75)	Non-recurrence (*n *= 150)	Statistic	*p*-value
Gender [*n* (%)]				10.449	0.001
	Male	31 (41.3%)	96 (64.0%)		
	Female	44 (58.7%)	54 (36.0%)		
BMI (mean ± standard deviation)		21.97 ± 6.04	21.77 ± 5.96	0.239	0.812
Educational status [*n* (%)]				4.069	0.254
	Primary school and below	26 (34.7%)	35 (23.3%)		
	Junior high school	19 (25.3%)	36 (24.0%)		
	High school or technical secondary school	17 (22.7%)	44 (29.3%)		
	College degree or above	13 (17.3%)	35 (23.3%)		
Marital status [*n* (%)]				4.844	0.089
	Married	30 (40.0%)	47 (31.3%)		
	Single	27 (36.0%)	45 (30.0%)		
	Divorce or widowed	18 (24.0%)	58 (38.7%)		
Family genetic background [*n* (%)]				1.389	0.239
	Yes	31 (41.3%)	50 (33.3%)		
	No	44 (58.7%)	100 (66.7%)		
Smoking status [*n* (%)]				1.282	0.258
	Yes	40 (53.3%)	68 (45.3%)		
	No	35 (46.7%)	82 (54.7%)		
Drinking status [*n* (%)]				5.363	0.021
	Yes	32 (42.7%)	41 (27.3%)		
	No	43 (57.3%)	109 (72.7%)		
Medication status [*n* (%)]				20.930	<0.001
	Regular medication	27 (36.0%)	102 (68.0%)		
	Irregular medication	48 (64.0%)	48 (32.0%)		
Premorbid personality [*n *(%)]				3.249	0.071
	Introversion	48 (64.0%)	77 (51.3%)		
	Extroversion	27 (36.0%)	73 (48.7%)		
Exercise frequency [*n* (%)]				21.363	<0.001
	<Once a week	55 (73.3%)	61 (40.7%)		
	≥Once a week	20 (26.7%)	89 (59.3%)		
Drug adverse reactions [*n* (%)]				11.571	0.001
	No	28 (37.3%)	92 (61.3%)		
	Yes	47 (62.7%)	58 (38.7%)		
Household registration type [*n* (%)]				1.280	0.258
	Urban	34 (45.3%)	80 (53.3%)		
	Rural	41 (54.7%)	70 (46.7%)		
Per capita monthly income [*n* (%)]				0.202	0.904
	≤2000 CNY	27 (36.0%)	58 (38.7%)		
	2000–4300 CNY	26 (34.7%)	48 (32.0%)		
	≥4300 CNY	22 (29.3%)	44 (29.3%)		
Payment method [*n *(%)]				0.326	0.850
	Employee insurance	23 (30.7%)	46 (30.7%)		
	Resident insurance	31 (41.3%)	57 (38.0%)		
	Self-payment	21 (28.0%)	47 (31.3%)		
Family care (mean ± standard deviation, score)		5.24 ± 2.23	6.83 ± 2.04	5.327	<0.001
Social support (mean ± standard deviation, score)		31.31 ± 10.31	35.15 ± 10.09	2.675	0.008
Life events (mean ± standard deviation, score)		12.61 ± 4.18	8.15 ± 3.55	8.376	<0.001
Sleep duration (mean ± standard deviation, hours)		5.08 ± 2.12	7.13 ± 1.82	7.531	<0.001

BMI, body mass index. $1.00 USD = ¥7.26 CNY.

### 3.3 Multivariate Logistic Regression Analysis

Multivariate logistic regression analysis employed recurrence status (recurrence = 1, non-recurrence = 0) as the dependent variable and statistically significant variables (*p* < 0.05) from univariate screening as covariates. With an introduction level of α input = 0.05 and removal level of α output = 0.10, collinearity was excluded based on variable VIF values ranging from 1.067 to 1.227. Results revealed: Irregular medication (*OR = *3.302,* 95% CI*: 1.386–7.871), low exercise frequency (*OR = *2.770,* 95% CI*: 1.141–6.726), family care (*OR = *0.647,* 95% CI*: 0.514–0.813), life events (*OR = *1.353,* 95% CI*: 1.194–1.533) and sleep duration (*OR = *0.630,* 95% CI*: 0.504–0.788) as independent influencing factors for recurrence in elderly patients undergoing maintenance phase treatment for SCZ (Table 3).

**Table 3.** **Multivariate regression and forest plot of factors affecting relapse in elderly schizophrenia patients during maintenance phase**.

Variables	B	SE	Wald χ^2^	OR (95% CI)	*p*-value	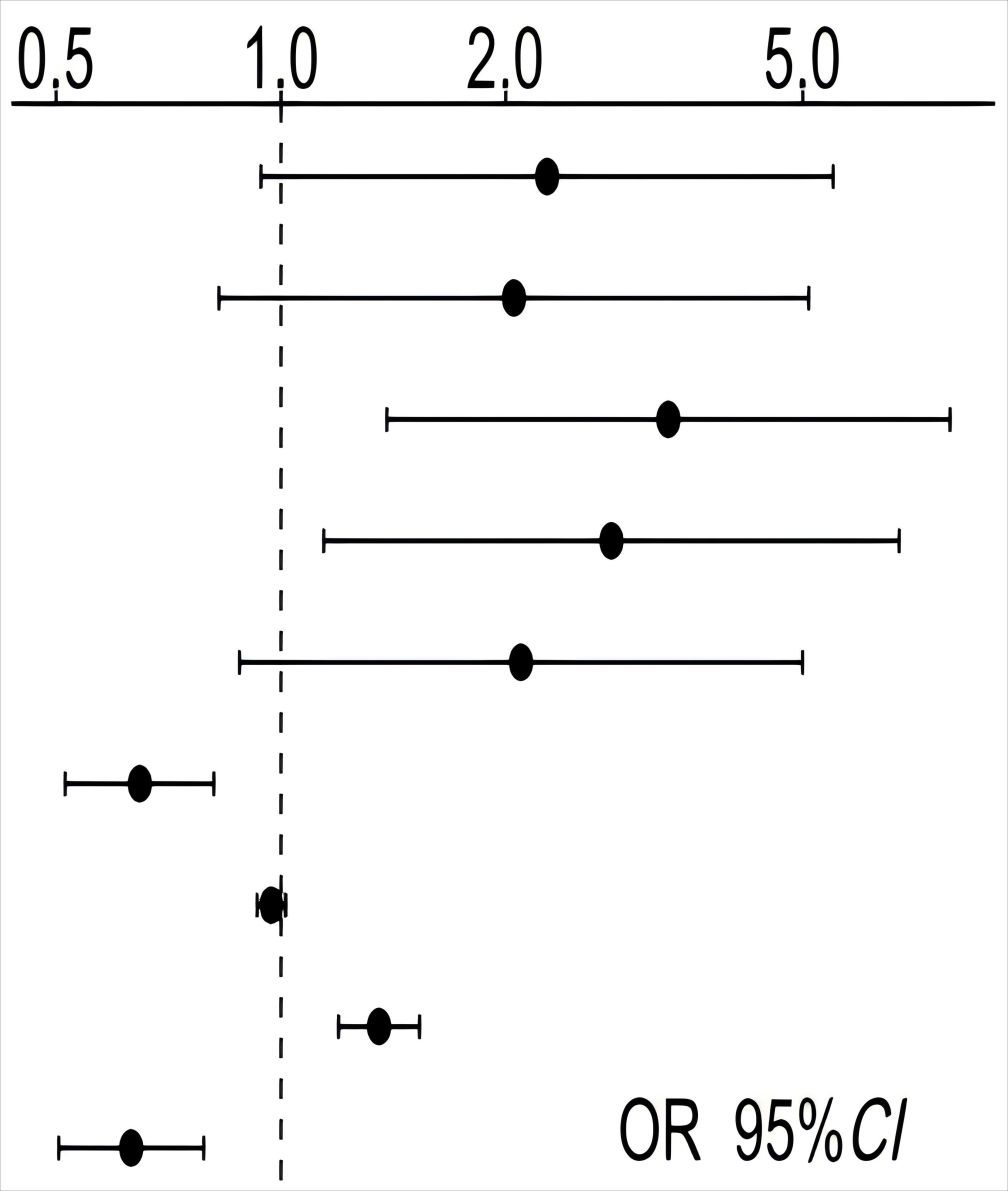
Sex (Female)	0.820	0.450	3.323	2.272 (0.940~5.488)	0.068	
Drinking status (YES)	0.718	0.464	2.397	2.051 (0.826~5.090)	0.122	
Medication status (Irregular)	1.195	0.443	7.269	3.302 (1.386~7.871)	0.007	
Exercise frequency (<Once a week)	1.019	0.453	5.069	2.770 (1.141~6.726)	0.024	
Drug adverse reactions (YES)	0.740	0.443	2.790	2.096 (0.880~4.994)	0.095	
Family care	–0.436	0.117	13.901	0.647 (0.514~0.813)	<0.001	
Social support	–0.029	0.023	1.654	0.971 (0.929~1.015)	0.198	
Life events	0.302	0.064	22.383	1.353 (1.194~1.533)	<0.001	
Sleep duration	–0.462	0.114	16.424	0.630 (0.504~0.788)	<0.001	

SE, standard error; OR, odds ratio; CI, confidence interval.

### 3.4 ROC Curves Analysis

**Fig. 1.**
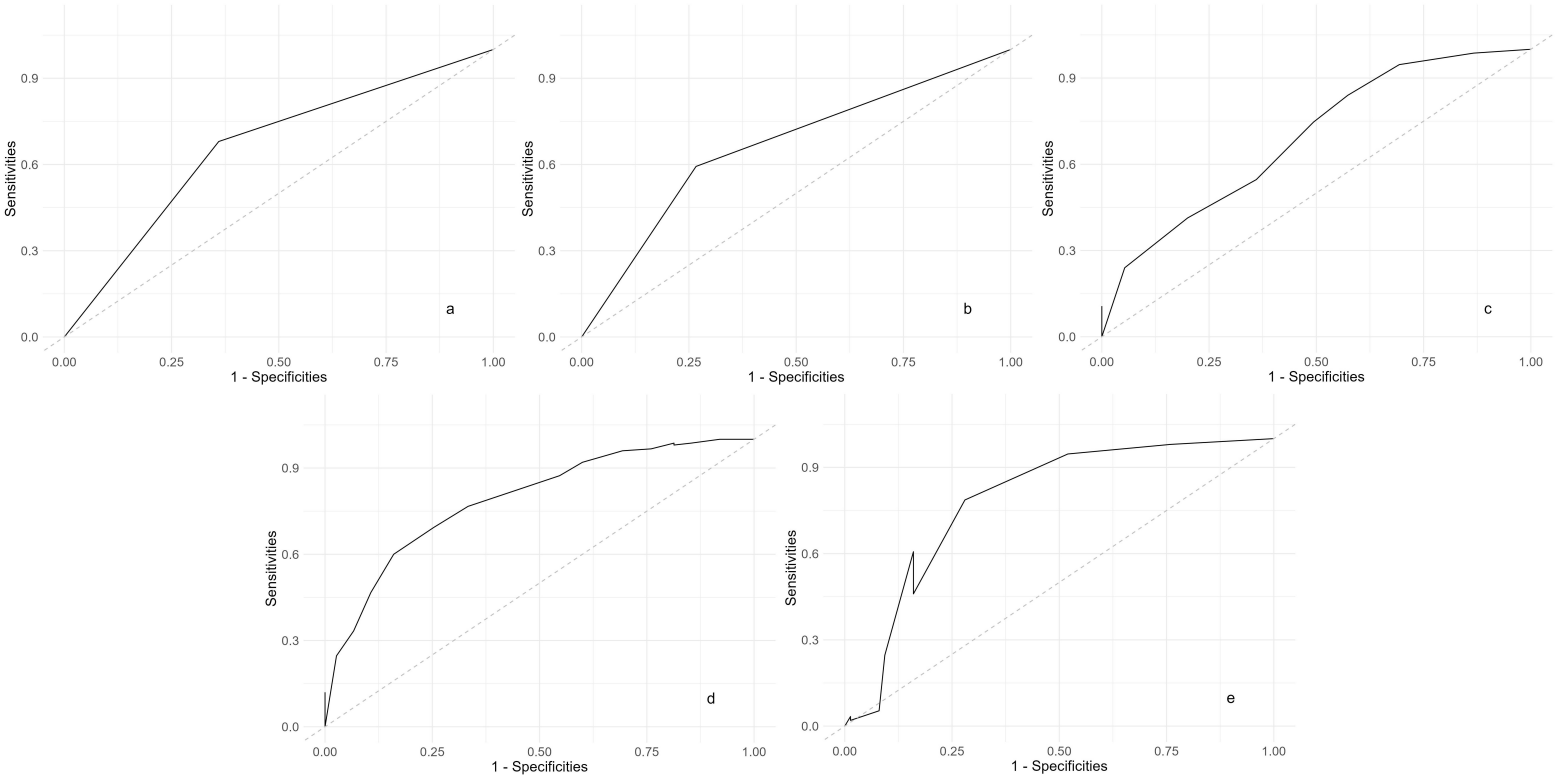
**ROC curves of all influencing factors distinguishing recurrence in elderly SCZ patients during the maintenance period**. (a) Medication status: ROC curve showing the predictive ability of medication adherence. (b) Exercise frequency: ROC curve showing the predictive ability of regular exercise. (c) Family care: ROC curve showing the impact of family support. (d) Life events: ROC curve showing the influence of significant life events. (e) Sleep duration: ROC curve showing the role of adequate sleep duration. ROC, the receiver operating characteristic; SCZ, schizophrenia.

**Fig. 2.**
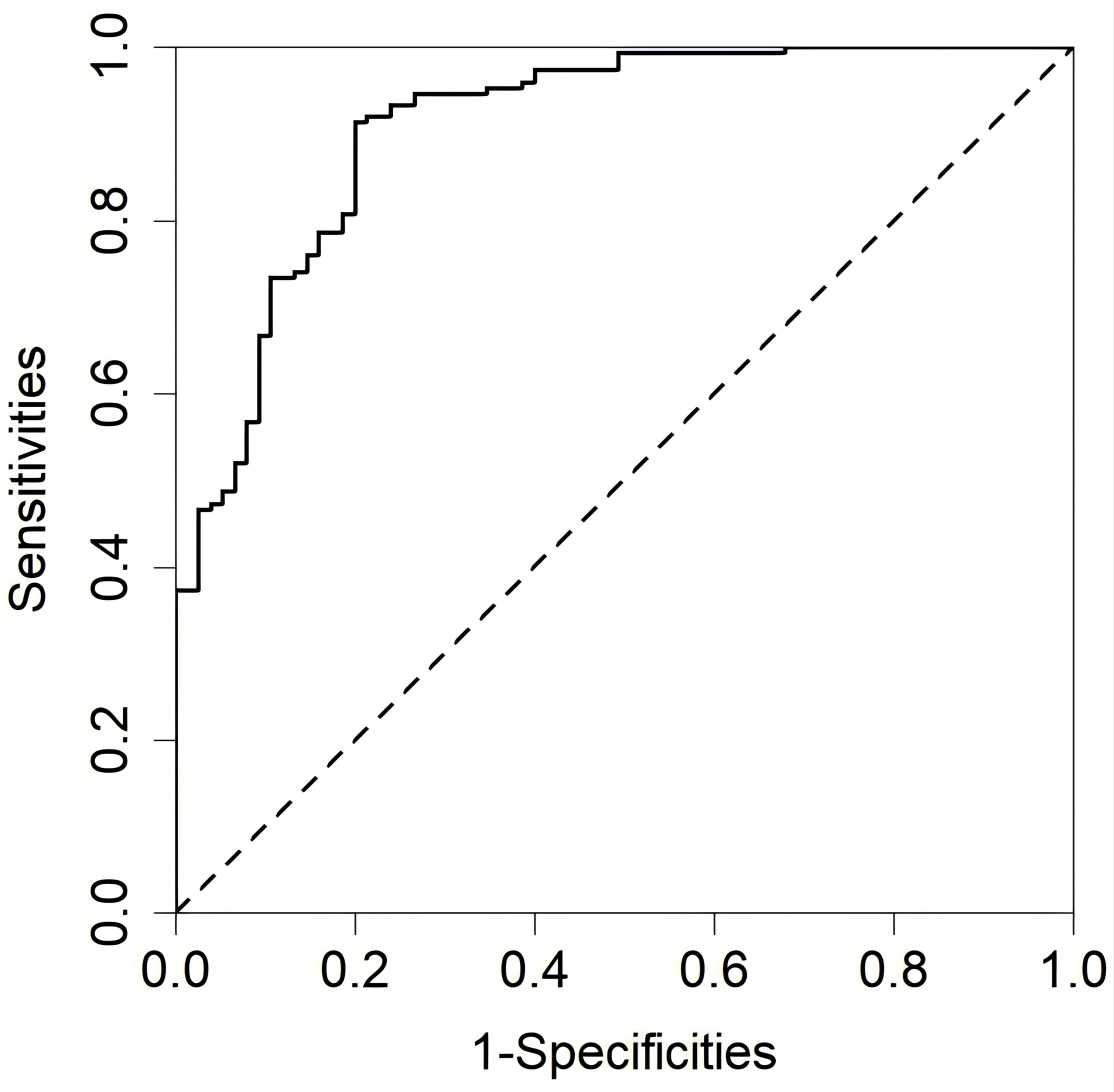
**Combined ROC curve of all influencing factors distinguishing recurrence in elderly SCZ patients during the maintenance period**.

Based on the results of the multivariate logistic regression analysis, ROC curves were constructed and the AUC was calculated to assess the predictive value of the influencing factors for relapse among elderly SCZ patients during maintenance phase treatment. ROC curves were separately generated for: Medication status (Fig. 1a), exercise frequency (Fig. 1b), family care (Fig. 1c), life events (Fig. 1d) and sleep duration (Fig. 1e) to differentiate relapse among elderly SCZ patients during maintenance phase treatment, as well as the ROC curve after incorporating all influencing factors (Fig. 2), with comparisons made (Table 4). When compared to the predictive value of individual influencing factors for relapse among elderly SCZ patients during maintenance phase treatment, the AUC after incorporating all influencing factors was 0.908 (*95% CI*: 0.867–0.949), indicating superior predictive value.

**Table 4.** **Comparison of ROC of recurrence in elderly schizophrenia patients during the maintenance period distinguished by various factors**.

Fig.	Influencing factor	AUC	95% CI	Threshold	Sensitivities	Specificities	Youden index
Fig. 1a	Medication status	0.660	0.594~0.726	0.665	0.680	0.640	0.320
Fig. 1b	Exercise frequency	0.663	0.599~0.727	0.798	0.593	0.733	0.326
Fig. 1c	Family care	0.691	0.618~0.764	0.164	0.840	0.164	0.004
Fig. 1d	Life events	0.792	0.731~0.853	0.899	0.693	0.746	0.439
Fig. 1e	Sleep duration	0.789	0.718~0.859	0.386	0.786	0.720	0.506
Fig. 2	All factors	0.908	0.867~0.949	0.176	0.913	0.800	0.713

ROC, receiver operating characteristic; AUC, area under the curve; CI, confidence interval.

## 4. Discussion

The findings of this study highlight several key factors influencing relapse among elderly patients with SCZ during maintenance therapy, including medication status, exercise frequency, family care, life events and sleep duration. Previous literature on relapse factors in SCZ patients has often overlooked variables such as exercise frequency and family care, which may underscore the unique risks faced by elderly patients during maintenance. Addressing these factors collectively may offer a more effective approach to preventing relapse in this population.

Medication status plays a crucial role in the relapse of elderly SCZ patients during maintenance phase treatment. In this study, the older age of the followed-up patients often led to instances of missed medication due to lack of familial support and declining memory. Additionally, as bodily functions deteriorate and metabolic rates decrease, the adverse effects of medication become more pronounced, indirectly reducing patients’ adherence to medication regimes. This reduction in adherence leads to adjustments in maintenance dosages required to promote disease stability, ultimately resulting in disease relapse. When treating elderly SCZ patients, it is preferable to adhere to the principles of single-drug therapy and low dosages [28], thereby avoiding issues of drug tolerance and improving medication status.

Exercise frequency is an important factor in the relapse of elderly SCZ patients during maintenance phase treatment. On one hand, as a supplement to medication therapy, regular exercise can reduce the severity of negative symptoms in patients and promote improvements in social functioning and quality of life [12]. Additionally, appropriate exercise facilitates the restoration of self-awareness, enabling patients to assess their own psychological condition and adopt appropriate coping strategies, thereby reducing the risk of disease relapse. On the other hand, from the perspective of the mechanism of electroencephalogram generation, exercise also plays a role in emotional regulation. Studies have shown that exercise inhibits activation in the right prefrontal cortex, reducing the generation of negative emotions and promoting the generation of more positive emotions, which impacts disease relapse [29]. Therefore, elderly SCZ patients should engage in appropriate exercise, such as aerobic exercise [30], under conditions that meet their physical requirements, to reduce the rate of disease relapse.

Family care is one of the influencing factors for relapse in elderly SCZ patients during maintenance phase treatment and it is also one of the most easily overlooked. The family is an emotional unit, a system where family members are interconnected and interdependent. Due to the older age of the patients followed in this study, sensitivity to changes in family care or the absence of family members, limited visiting or companionship time from children, or even their absence, may lead to dysfunction within the family, where inner emotions and desires cannot be expressed, resulting in emotional fluctuations that affect disease relapse. Good family care is beneficial for both treatment adherence and the improvement of anxiety in patients, which helps maintain the patient in a relatively stable state.

Life events constitute another significant factor influencing relapse, particularly negative events. Inadequate coping with adverse life events, characterized by inaccurate assessment, maladaptive solutions and unaddressed negative emotions, precipitate emotional upheaval, thereby heightening relapse risk. Consequently, implementing appropriate strategies to guide patients in navigating life stressors is imperative for relapse prevention.

The duration of sleep is also associated with disease relapse. Sleep disturbances serve as stressors, triggering physiological stress responses and mood fluctuations, thereby exacerbating relapse vulnerability [31]. Creating conducive sleep environments and maintaining emotional equilibrium are essential strategies for extending sleep duration and reducing relapse rates.

## 5. Limitations of the Study

This study considers exercise status as a factor influencing relapse. However, compared to other age groups, a decrease in the willingness of elderly SCZ patients to exercise or a decline or loss of physical ability due to physical illnesses may introduce sampling errors and affect the results. Therefore, this factor should be taken into account when developing strategies to address relapse. The comprehensiveness of the conclusions may be affected by the limitations of the hospital information system. Furthermore, as this study’s sample is derived from a single-center retrospective study, which may introduce biases in exploring the impact of relapse influenced by factors such as different cultural levels and regional economic status. Missing data were handled by revisitation to gather information, but the accuracy of recall may have been affected by time constraints. Future research could involve multi-center controlled trials to further determine the factors influencing relapse in elderly SCZ patients during the maintenance period.

## 6. Conclusion

Addressing the multifaceted factors explored here collectively offers a promising approach to enhance relapse prevention strategies in elderly patients with schizophrenia. In terms of treatment, a principle of using single medications at low doses is recommended to maintain symptom stability, minimize medication side effects and enhance medication status. Within the constraints of physical health, appropriate exercise, particularly aerobic exercise, should be encouraged to facilitate emotional expression and increase family support, thereby enhancing patient ability to cope with life events and creating conducive environments to improve sleep duration. Implementing integrated and effective measures can effectively reduce the recurrence rate of SCZ.

## Availability of Data and Materials

The raw data supporting the conclusions of this article will be made available by the authors, without undue reservation. Those seeking data and materials, please contact the corresponding author: lwt0411@sina.com.

## Appendix

See Table 5.

**Appendix Table 5.** **Variable assignment methods for univariate regression**.

Factors	Type of variable	Quantification method
Gender	categorical variables	Male = 1, Female = 2
BMI	continuous variables	
Educational status	categorical variables	Primary school and below = 1, Junior high school = 2, High school or technical secondary school = 3, College degree or above = 4
Marital status	categorical variables	Married = 1, Single = 2, Divorce or Widowed = 3
Family genetic background	categorical variables	No = 0, Yes = 1
Smoking status	categorical variables	No = 0, Yes = 1
Drinking status	categorical variables	No = 0, Yes = 1
Premorbid personality	categorical variables	Introversion = 1, Extroversion = 2
Exercise frequency	categorical variables	<Once a week = 1, ≥Once a week = 2
Drug adverse reactions	categorical variables	No = 0, Yes = 1
Household registration type	categorical variables	Urban = 1, Rural = 2
Per capita monthly income (CNY)	categorical variables	≤2000 = 1, 2000–4300 = 2, ≥4300 = 3
Payment method	categorical variables	Employee insurance = 1, Resident insurance = 2, Self-payment = 3
Medication status	categorical variables	Irregular medication = 1, Regular medication = 2
Family care	continuous variables	
Social support	continuous variables	
Life events	continuous variables	
Sleep duration	continuous variables	

$1.00 USD = ¥7.26 CNY.
